# Supplementary material for: Diet-Wide Association Study for the Incidence of Type 2 Diabetes Mellitus in Community-Dwelling Adults Using the UK Biobank Data
Source: Nutrients. 2023 Dec 28;16(1):103. doi: 10.3390/nu16010103 (PMC10780379; doi:10.3390/nu16010103)
Supplement: Supplementary file 1 [file nutrients-16-00103-s001.zip › nutrients-2673200-supplementary.pdf]

Supplementary Information

Table S1. Dietary foods and nutrients information.

Figure S1. Forest plot for the association between dietary food and nutrient factors and T2DM risk in Model 1-3.

Figure S2. Sensitivity analysis for subjects who participated ≥3 times dietary assessments.

Figure S3. Sensitivity analysis excluding participants with nutrient intake among the top/bottom 1%.

Figure S4. Kaplan-Meier survival curves.

Table S1a. Dietary food information.

Category	Variable (Unit)
Grain	Couscous (Surving)
Grain	Other cooked grains (e.g. bulgur wheat, millet or pearl barley) (Serving)
Grain	Rice (White) (Serving)
Grain	Rice (Brown) (Serving)
Grain	Snack pot (Serving)
Grain	Sushi (Sushi)
Grain	Pasta (White) (Serving)
Grain	Pasta (Wholemeal) (Serving)
Grain	Bran cereals (bowl)
Grain	Muesli (bowl)
Grain	Oat cereal (bowl)
Grain	Other cereal (bowl)
Grain	Other sweetened cereals (bowl)
Grain	Plain cereals (bowl)
Grain	Porridge (bowl)
Grain	Wholewheat cereals (bowl)
Grain	Baguette (Mixed) (Bread item)
Grain	Baguette (Other) (Bread item)
Grain	Baguette (Seeded) (Bread item)
Grain	Baguette (White) (Bread item)
Grain	Baguette (Wholemeal) (Bread item)
Grain	Bap (Mixed) (Bread item)
Grain	Bap (Other) (Bread item)
Grain	Bap (Seeded) (Bread item)
Grain	Bap (White) (Bread item)
Grain	Bap (Wholemeal) (Bread item)
Grain	Bread roll (Mixed) (Bread item)
Grain	Bread roll (Other) (Bread item)
Grain	Bread roll (Seeded) (Bread item)
Grain	Bread roll (White) (Bread item)
Grain	Bread roll (Wholemeal) (Bread item)
Grain	Sliced bread (Mixed) (Slice)
Grain	Sliced bread (Other) (Bread item)
Grain	Sliced bread (Seeded) (Bread item)
Grain	Sliced bread (White) (Bread item)
Grain	Sliced bread (Wholemeal) (Bread item)
Grain	Baguette (Butter) (Bread item)
Grain	Bap (Butter) (Bread item)
Grain	Bread roll (Butter) (Bread item)
Grain	Crackers (Butter) (Biscuit)
Grain	Crispbread (Biscuit/item)
Grain	Garlic bread (Slice)
Grain	Naan bread (Bread item)
Grain	Oatcakes (Biscuit)
Grain	Oatcakes (Butter) (Biscuit)
Grain	Other bread (e.g. crumpets, tortilla wraps, breadsticks) (Slice/item)
Grain	Other bread (e.g. crumpets, tortilla wraps, breadsticks) (Butter) (Slice/item)

Grain	Slice bread (Butter) (Slice)
Vegetable	Avocado (Medium avocado)
Vegetable	Beans (baked beans) (Serving)
Vegetable	Beans (broad beans) (Serving)
Vegetable	Beans (green, French, runner) (Serving)
Vegetable	Beetroot (Serving)
Vegetable	Broccoli (Serving)
Vegetable	Butternut squash (Serving)
Vegetable	Cabbage, greens, kale (Serving)
Vegetable	Carrot (fresh, frozen, raw, cooked) (Serving)
Vegetable	Cauliflower (Serving)
Vegetable	Celery (Stick)
Vegetable	Coleslaw or other salad vegetables in mayonnaise (e.g. waldorf salad) (Serving)
Vegetable	Courgette (Serving)
Vegetable	Cucumber (1 inch/2.5cm)
Vegetable	Garlic (Clove)
Vegetable	Leeks (Serving)
Vegetable	Lettuce (Serving)
Vegetable	Mixed side salad (Serving)
Vegetable	Mixed vegetables (e.g. frozen mixed vegetable)
Vegetable	Mushrooms (Serving)
Vegetable	Onion (red, white, pickled, shallots, spring) (Serving)
Vegetable	Other vegetables (e.g. celeriac, asparagus, fennel, aubergine, pumpkin) (Serving)
Vegetable	Parsnip (Serving)
Vegetable	Peas (frozen, fresh, tinned) (Serving)
Vegetable	Peppers (Sweet) (Whole pepper)
Vegetable	Potatoes (fried, chips, wedges, roast) (Serving)
Vegetable	Potatoes (Baked, boiled) (Serving)
Vegetable	Potatoes (mashed) (Serving)
Vegetable	Pulses (Serving)
Vegetable	Spinach (Serving)
Vegetable	Sprouts (Serving)
Vegetable	Sweet potatoes (Serving)
Vegetable	Sweetcorn (tinned or frozen, or corn on the cob) (Serving)
Vegetable	Tomatoes (fresh) (Medium tomato)
Vegetable	Tomatoes (cooked or tinned) (Serving)
Vegetable	Turnip or swede (Serving)
Vegetable	Vegetable pieces (too small to be counted as individual vegetable) (Serving)
Vegetable	Watercress (Serving)
Vegan	Other vegetarian alternative (Serving)
Vegan	Quon (Serving)
Vegan	Tofu (Serving)
Vegan	Vegetarian sausage (Each)
Fruit	Apple (Whole fruit)
Fruit	Banana (Whole fruit)
Fruit	Berries (Handful)
Fruit	Cherries (Handful)
Fruit	Dried fruit (Handful)
Fruit	Grape (Handful)

Fruit	Grapefruit (Whole fruit/serving)
Fruit	Mango (Serving)
Fruit	Melon (Serving)
Fruit	Mixed fruit (Serving)
Fruit	Orange (Whole fruit)
Fruit	Orange-like small fruits e.g. satsuma, clementine, mandarin (Whole fruit/serving)
Fruit	Other fruit e.g. pomegranate, kiwi, papaya (Whole fruit/serving)
Fruit	Peach, nectarine (Whole fruit/serving)
Fruit	Pear (Whole fruit/serving)
Fruit	Pineapple (Serving)
Fruit	Plum (Whole fruit)
Fruit	Prunes (Handful)
Fruit	Stewed/cooked fruit e.g. apple, rhubarb, plums (Serving)
Pastries	Cake, muffin, flapjack, brownie, pecan pie (Slice/Item)
Pastries	Cheesecake (Serving)
Pastries	Croissant (Pastry)
Pastries	Crumble topping (Serving)
Pastries	Danish pastry (Pastry)
Pastries	Double crust pie (Slice/Item)
Pastries	Doughnuts (Item)
Pastries	Fruit cake (Slice)
Pastries	Ice cream (Serving/Individual ice cream)
Pastries	Indian snacks (Item)
Pastries	Milk pudding (Serving/Individual pot)
Pastries	Other dessert (Serving/Individual pot)
Pastries	Other milk based desserts (Serving/Individual pot)
Pastries	Pancake (Pancake)
Pastries	Pizza (Medium slice)
Pastries	Scone (Scone)
Pastries	Scotch pancake (Pancake)
Pastries	Single crust pastry (Slice/Item)
Pastries	Soya dessert (Serving/Individual pot)
Pastries	Sponge pudding (Serving)
Pastries	Yorkshire pudding (Muffin-sized pudding/Serving)
Snack	Ceral bars (Bar [~50g])
Snack	Cheesy biscuits (Handful/Bag)
Snack	Chocolate bar (Bar [~50g])
Snack	Chocolate biscuits (Biscuit)
Snack	Chocolate covered biscuits (Biscuit)
Snack	Chocolate covered raisins (Handful)
Snack	Chocolate sweets (Handful)
Snack	Crisps (Small bag)
Snack	Dark chocolate (Bar [~50g])
Snack	Diet sweets (Handful)
Snack	Milk chocolate (Bar [~50g])
Snack	Olives (Handful)
Snack	Other savoury snack (Handful/Bag)
Snack	Other sweets snack (Handful/Item)
Snack	Salted nuts (Handful)

Snack	Salted peanuts (Handful)
Snack	Savoury biscuits (Bag)
Snack	Seeds (Handful)
Snack	Sweet biscuits (Biscuit)
Snack	Sweets (Handful)
Snack	Unsalted nuts (Handful)
Snack	Unsalted peanuts (Handful)
Snack	White chocolate (Bar [~50g])
Soup	Canned soup (Bowl)
Soup	Home-made soup (Bowl)
Soup	Powered soup (Mug/Bowl)
Meat/fish	Bacon (Rasher)
Meat/fish	Beef (Serving)
Meat/fish	Ham (Slice)
Meat/fish	Lamb (Serving)
Meat/fish	Liver (Serving)
Meat/fish	Other meat (Serving)
Meat/fish	Pork (Serving)
Meat/fish	Poultry (Crumbed or deep-fried) (Serving)
Meat/fish	Poultry (Serving)
Meat/fish	Sausage (Each)
Meat/fish	Battered fish (Serving)
Meat/fish	Breaded fish (e.g. fish fingers) or fish cakes (Serving)
Meat/fish	Lobster or crab (Serving)
Meat/fish	Oily fish (e.g. salmon, tinned salmon, herring, mackerel, sardines, fresh tuna steak) (Serving)
Meat/fish	Other fish (Serving)
Meat/fish	Prawns (Serving)
Meat/fish	Shelfish (e.g. mussels, scallops) (Serving)
Meat/fish	Tinned tuna (Serving)
Meat/fish	White fish (e.g. cod, haddock, fish pie)
Diary product/eggs	Egg in sandwiches (Serving)
Diary product/eggs	Omelettes (Serving)
Diary product/eggs	Other egg dishes (Serving)
Diary product/eggs	Scotch egg (Each)
Diary product/eggs	Whole eggs (Number of eggs)
Diary product/eggs	Blue cheese (Serving)
Diary product/eggs	Cottage cheese (Serving)
Diary product/eggs	Feta (Serving)
Diary product/eggs	Goat cheese (Serving)
Diary product/eggs	Hard cheese (Serving)
Diary product/eggs	Low fat hard cheese (Serving)
Diary product/eggs	Low fat spreadable cheese (Serving)
Diary product/eggs	Milk (Excluding milkshakes) (Glass/Carton/250ml)
Diary product/eggs	Mozzarella (Serving)
Diary product/eggs	Other cheese (Serving)
Diary product/eggs	Soft cheese (Serving)
Diary product/eggs	Spreadable cheese (Serving)
Diary product/eggs	Yogurt (Serving/Individual pot)
Beverages	Green tea (Mug/Cup)

Beverages	Herbal or fruit tea (infusion) (Mug/Cup)
Beverages	Other tea/infusion (Mug/Cup)
Beverages	Rooibos/Redbush tea (e.g. Tick Tock) (Mug/Cup)
Beverages	Standard tea (e.g. Tetley, PG Tips, Assam, Darjeeling) (Mug/Cup)
Beverages	Cappuccino (Mug/Cup)
Beverages	Espresso (Cup)
Beverages	Filter coffee (Mug/Cup)
Beverages	Instant coffee (Mug/Cup)
Beverages	Latte (Mug/Cup)
Beverages	Other coffee drinks (Mug/Cup)
Beverages	Carbonated (fizzy) drinks (Glass/Can)
Beverages	Dairy/yogurt based smoothie (Glass/Bottle/250ml)
Beverages	Fruit smoothie (Glass/Bottle/250ml)
Beverages	Fruit drinks, J2O, squash or cordial (Glass/Carton/250ml)
Beverages	Hot chocolate or other milk-based drinks (including Horlicks) (Mug/Cup)
Beverages	Low calorie hot chocolate drinks (Mug/Cup)
Beverages	Low calorie or diet drinks (e.g. fizzy, squash) (Glass/Can)
Beverages	Other drinks (Glass/Mug/Cup)
Beverages	Other pure fruit/vegetable juice (Glass/Carton/250ml)
Beverages	Pure grapefruit juice (Glass/Carton/250ml)
Beverages	Pure orange juice (Glass/Carton/250ml)
Beverages	Water (Glass/Beaker)
Beverages	Yogurt drinks, flavoured milk or milkshakes (Glass/Carton/250ml)
Alcoholic beverages	Alcohol (ml)
Alcoholic beverages	Beer/cider (ml)
Alcoholic beverages	Fortified wine (ml)
Alcoholic beverages	Other alcohol drinks (ml)
Alcoholic beverages	Red wine (ml)
Alcoholic beverages	Rose wine (ml)
Alcoholic beverages	Spirits (Measure)
Alcoholic beverages	White wine (ml)

Table S1b. Dietary nutrient information.

Variable (unit)
Calcium (mg/day)
Carbohydrate (% of energy intake/day)
Carotene (µg/day)
Energy (kj/day)
Fat (% of energy intake/day)
Fibre (g/day)
Folate (µg/day)
Iron (mg/day)
Magnesium (mg/day)
Potassium (mg/day)
Protein (% of energy intake/day)
Polyunsaturated fat (% of energy intake/day)
Retinol (µg/day)
Saturated fatty acids (% of energy intake/day)
Starch (g/day)
Sugar (g/day)
Vitamin B12 (mg/day)
Vitamin B6 (mg/day)
Vitamin C (mg/day)
Vitamin D (µg/day)
Vitamin E (mg/day)

Figure S1. Forest plot for the association between dietary food and nutrient factors and T2DM risk in Model 1-3.

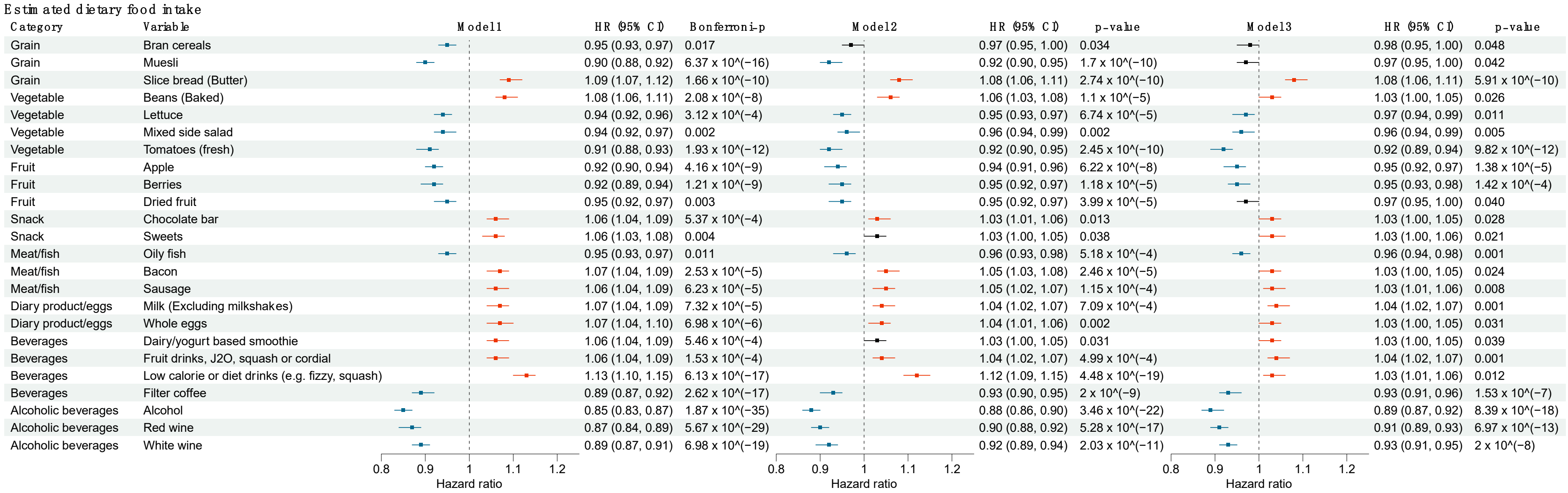

Estimated dietary food intake

Category	Variable	Model1 HR (95% CI)	Bonferroni-p	Model2 HR (95% CI)	p-value	Model3 HR (95% CI)	p-value
Grain	Bran cereals	0.95 (0.93, 0.97)	0.017	0.97 (0.95, 1.00)	0.034	0.98 (0.95, 1.00)	0.048
Grain	Muesli	0.90 (0.88, 0.92)	6.37 x 10^(−16)	0.92 (0.90, 0.95)	1.7 x 10^(−10)	0.97 (0.95, 1.00)	0.042
Grain	Slice bread (Butter)	1.09 (1.07, 1.12)	1.66 x 10^(−10)	1.08 (1.06, 1.11)	2.74 x 10^(−10)	1.08 (1.06, 1.11)	5.91 x 10^(−10)
Vegetable	Beans (Baked)	1.08 (1.06, 1.11)	2.08 x 10^(−8)	1.06 (1.03, 1.08)	1.1 x 10^(−5)	1.03 (1.00, 1.05)	0.026
Vegetable	Lettuce	0.94 (0.92, 0.96)	3.12 x 10^(−4)	0.95 (0.93, 0.97)	6.74 x 10^(−5)	0.97 (0.94, 0.99)	0.011
Vegetable	Mixed side salad	0.94 (0.92, 0.97)	0.002	0.96 (0.94, 0.99)	0.002	0.96 (0.94, 0.99)	0.005
Vegetable	Tomatoes (fresh)	0.91 (0.88, 0.93)	1.93 x 10^(−12)	0.92 (0.90, 0.95)	2.45 x 10^(−10)	0.92 (0.89, 0.94)	9.82 x 10^(−12)
Fruit	Apple	0.92 (0.90, 0.94)	4.16 x 10^(−9)	0.94 (0.91, 0.96)	6.22 x 10^(−8)	0.95 (0.92, 0.97)	1.38 x 10^(−5)
Fruit	Berries	0.92 (0.89, 0.94)	1.21 x 10^(−9)	0.95 (0.92, 0.97)	1.18 x 10^(−5)	0.95 (0.93, 0.98)	1.42 x 10^(−4)
Fruit	Dried fruit	0.95 (0.92, 0.97)	0.003	0.95 (0.92, 0.97)	3.99 x 10^(−5)	0.97 (0.95, 1.00)	0.040
Snack	Chocolate bar	1.06 (1.04, 1.09)	5.37 x 10^(−4)	1.03 (1.01, 1.06)	0.013	1.03 (1.00, 1.05)	0.028
Snack	Sweets	1.06 (1.03, 1.08)	0.004	1.03 (1.00, 1.05)	0.038	1.03 (1.00, 1.06)	0.021
Meat/fish	Oily fish	0.95 (0.93, 0.97)	0.011	0.96 (0.93, 0.98)	5.18 x 10^(−4)	0.96 (0.94, 0.98)	0.001
Meat/fish	Bacon	1.07 (1.04, 1.09)	2.53 x 10^(−5)	1.05 (1.03, 1.08)	2.46 x 10^(−5)	1.03 (1.00, 1.05)	0.024
Meat/fish	Sausage	1.06 (1.04, 1.09)	6.23 x 10^(−5)	1.05 (1.02, 1.07)	1.15 x 10^(−4)	1.03 (1.01, 1.06)	0.008
Diary product/eggs	Milk (Excluding milkshakes)	1.07 (1.04, 1.09)	7.32 x 10^(−5)	1.04 (1.02, 1.07)	7.09 x 10^(−4)	1.04 (1.02, 1.07)	0.001
Diary product/eggs	Whole eggs	1.07 (1.04, 1.10)	6.98 x 10^(−6)	1.04 (1.01, 1.06)	0.002	1.03 (1.00, 1.05)	0.031
Beverages	Dairy/yogurt based smoothie	1.06 (1.04, 1.09)	5.46 x 10^(−4)	1.03 (1.00, 1.05)	0.031	1.03 (1.00, 1.05)	0.039
Beverages	Fruit drinks, J2O, squash or cordial	1.06 (1.04, 1.09)	1.53 x 10^(−4)	1.04 (1.02, 1.07)	4.99 x 10^(−4)	1.04 (1.02, 1.07)	0.001
Beverages	Low calorie or diet drinks (e.g. fizzy, squash)	1.13 (1.10, 1.15)	6.13 x 10^(−17)	1.12 (1.09, 1.15)	4.48 x 10^(−19)	1.03 (1.01, 1.06)	0.012
Beverages	Filter coffee	0.89 (0.87, 0.92)	2.62 x 10^(−17)	0.93 (0.90, 0.95)	2 x 10^(−9)	0.93 (0.91, 0.96)	1.53 x 10^(−7)
Alcoholic beverages	Alcohol	0.85 (0.83, 0.87)	1.87 x 10^(−35)	0.88 (0.86, 0.90)	3.46 x 10^(−22)	0.89 (0.87, 0.92)	8.39 x 10^(−18)
Alcoholic beverages	Red wine	0.87 (0.84, 0.89)	5.67 x 10^(−29)	0.90 (0.88, 0.92)	5.28 x 10^(−17)	0.91 (0.89, 0.93)	6.97 x 10^(−13)
Alcoholic beverages	White wine	0.89 (0.87, 0.91)	6.98 x 10^(−19)	0.92 (0.89, 0.94)	2.03 x 10^(−11)	0.93 (0.91, 0.95)	2 x 10^(−8)

(a)

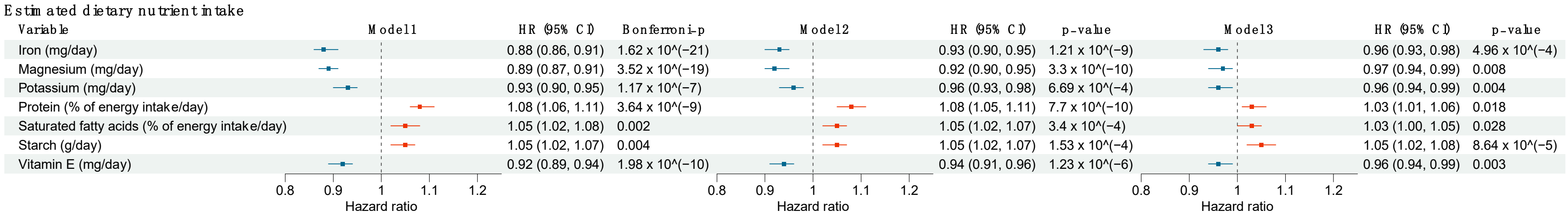

Estimated dietary nutrient intake

Variable	Model1 HR (95% CI)	Bonferroni-p	Model2 HR (95% CI)	p-value	Model3 HR (95% CI)	p-value
Iron (mg/day)	0.88 (0.86, 0.91)	1.62 x 10^(−21)	0.93 (0.90, 0.95)	1.21 x 10^(−9)	0.96 (0.93, 0.98)	4.96 x 10^(−4)
Magnesium (mg/day)	0.89 (0.87, 0.91)	3.52 x 10^(−19)	0.92 (0.90, 0.95)	3.3 x 10^(−10)	0.97 (0.94, 0.99)	0.008
Potassium (mg/day)	0.93 (0.90, 0.95)	1.17 x 10^(−7)	0.96 (0.93, 0.98)	6.69 x 10^(−4)	0.96 (0.94, 0.99)	0.004
Protein (% of energy intake/day)	1.08 (1.06, 1.11)	3.64 x 10^(−9)	1.08 (1.05, 1.11)	7.7 x 10^(−10)	1.03 (1.01, 1.06)	0.018
Saturated fatty acids (% of energy intake/day)	1.05 (1.02, 1.08)	0.002	1.05 (1.02, 1.07)	3.4 x 10^(−4)	1.03 (1.00, 1.05)	0.028
Starch (g/day)	1.05 (1.02, 1.07)	0.004	1.05 (1.02, 1.07)	1.53 x 10^(−4)	1.05 (1.02, 1.08)	8.64 x 10^(−5)
Vitamin E (mg/day)	0.92 (0.89, 0.94)	1.98 x 10^(−10)	0.94 (0.91, 0.96)	1.23 x 10^(−6)	0.96 (0.94, 0.99)	0.003

(b)

Figure S1. Forest plot for the association between dietary food factors and T2DM risk (a) and the association between dietary nutrient factors and T2DM risk (b) in Model 1-3. Abbreviation: HR, hazard ratio; CI, confidence interval. The HR represents the increased T2DM risk for each quintile increase in dietary factor consumption. Model 1 is adjusted for gender and age at recruitment. Model 2 is adjusted for ethnic background, smoking

status, physical activity, education level, and Townsend index. Model 3 is further adjusted for BMI, HbA1c, blood cholesterol, medication for blood pressure and exogenous hormone, and medical condition of hypertension. For Models 2 and 3, non-alcoholic dietary food and nutrient variables were additionally adjusted for alcohol intake. Negative associations are depicted in blue, while positive associations are shown in red. Factors that are significant in both Model 1 and Model 2 will be highlighted in Model 2. Factors that are significant in all models will be highlighted in the Model 3 column. Negative associations are depicted in blue, while positive associations are shown in red.

Figure S2. Sensitivity analysis for subjects who participated ≥3 times dietary assessments.

S2a)

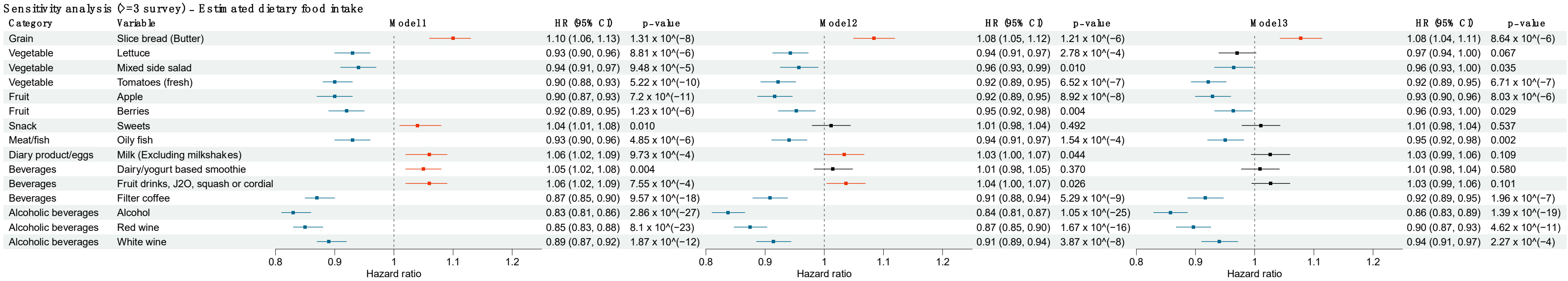

S2b)

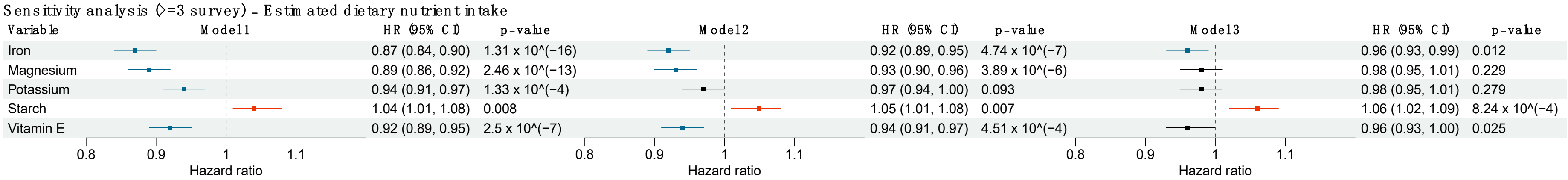

Figure S2. Forest plot for the association between significant dietary nutrient factors and T2DM risk (S2a) and the association between significant dietary nutrient factors and T2DM risk (S2b) for subjects who participated ≥3 times dietary assessments. Abbreviation: HR, Hazard ratio; CI, confidence interval. The HR represents the increased T2DM risk for each quintile increase in dietary factor consumption. Model 1 is adjusted for gender and age at recruitment. Model 2 is additionally adjusted for ethnic background, smoking status, physical activity, education level, and Townsend index. Model 3 is further adjusted for BMI, HbA1c, blood cholesterol, medication for blood pressure and exogenous hormone, and medical condition of hypertension. For Model 2 and 3, non-alcoholic dietary food and nutrient variables were additionally adjusted for alcohol intake. Negative associations are depicted in blue, while positive associations are shown in red. Factors that are significant in both Model 1 and Model 2 will be highlighted in Model 2. Factors that are significant in all models will be highlighted in the Model 3 column.

Figure S3. Sensitivity analysis excluding participants with nutrient intake among the top and bottom 1%.

S3a)

Sensitivity analysis (99%) - Estimated dietary food intake

Category	Variable	Model1 HR (95% CI)	Model1 p-value	Model2 HR (95% CI)	Model2 p-value	Model3 HR (95% CI)	Model3 p-value
Grain	Slice bread (Butter)	1.10 (1.07, 1.12)	3.76 x 10^(−13)	1.09 (1.06, 1.11)	1.1 x 10^(−10)	1.08 (1.06, 1.11)	3.28 x 10^(−10)
Vegetable	Lettuce	0.94 (0.92, 0.97)	3.04 x 10^(−6)	0.95 (0.93, 0.98)	1.49 x 10^(−4)	0.97 (0.95, 1.00)	0.019
Vegetable	Mixed side salad	0.95 (0.92, 0.97)	1.91 x 10^(−5)	0.96 (0.94, 0.99)	0.003	0.97 (0.94, 0.99)	0.007
Vegetable	Tomatoes (fresh)	0.91 (0.89, 0.93)	8.37 x 10^(−14)	0.93 (0.90, 0.95)	1.65 x 10^(−9)	0.92 (0.90, 0.95)	6.87 x 10^(−10)
Fruit	Apple	0.92 (0.90, 0.95)	1 x 10^(−10)	0.94 (0.91, 0.96)	2.46 x 10^(−7)	0.95 (0.93, 0.97)	3.6 x 10^(−5)
Fruit	Berries	0.91 (0.89, 0.94)	2.54 x 10^(−12)	0.94 (0.92, 0.97)	7.93 x 10^(−6)	0.95 (0.93, 0.98)	1.02 x 10^(−4)
Meat/fish	Oily fish	0.95 (0.93, 0.97)	5.96 x 10^(−5)	0.96 (0.93, 0.98)	6.82 x 10^(−4)	0.96 (0.94, 0.98)	0.001
Diary product/eggs	Milk (Excluding milkshakes)	1.06 (1.04, 1.09)	1.28 x 10^(−6)	1.04 (1.01, 1.07)	0.002	1.04 (1.01, 1.06)	0.003
Beverages	Dairy/yogurt based smoothie	1.06 (1.04, 1.09)	2.82 x 10^(−6)	1.03 (1.00, 1.05)	0.028	1.03 (1.00, 1.05)	0.032
Beverages	Fruit drinks, J2O, squash or cordial	1.07 (1.04, 1.09)	2.89 x 10^(−7)	1.05 (1.02, 1.07)	2.42 x 10^(−4)	1.04 (1.02, 1.07)	6.88 x 10^(−4)
Beverages	Filter coffee	0.89 (0.87, 0.91)	8.03 x 10^(−21)	0.92 (0.90, 0.95)	3.83 x 10^(−10)	0.93 (0.91, 0.95)	2.97 x 10^(−8)
Alcoholic beverages	Alcohol	0.87 (0.85, 0.89)	5.37 x 10^(−28)	0.87 (0.85, 0.89)	8.44 x 10^(−28)	0.88 (0.86, 0.90)	9.46 x 10^(−23)
Alcoholic beverages	Red wine	0.89 (0.86, 0.91)	5.05 x 10^(−23)	0.90 (0.88, 0.92)	4.73 x 10^(−17)	0.91 (0.89, 0.94)	1.84 x 10^(−12)
Alcoholic beverages	White wine	0.90 (0.87, 0.92)	9.18 x 10^(−18)	0.91 (0.88, 0.93)	4.81 x 10^(−14)	0.92 (0.89, 0.94)	2.12 x 10^(−11)

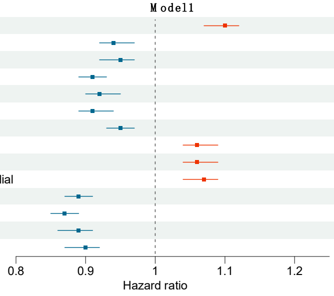

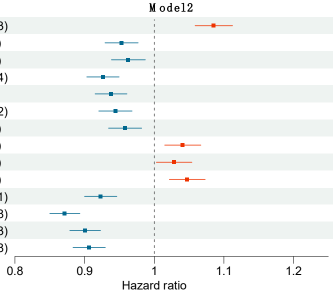

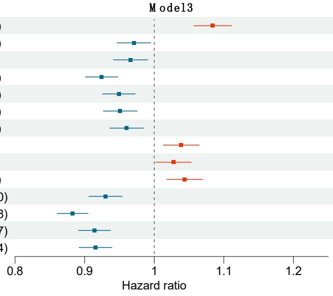

S3b)

Sensitivity analysis (99%) - Estimated dietary nutrient intake

Variable	Model1 HR (95% CI)	Model1 p-value	Model2 HR (95% CI)	Model2 p-value	Model3 HR (95% CI)	Model3 p-value
Iron	0.88 (0.86, 0.91)	1.26 x 10^(−22)	0.92 (0.90, 0.95)	8.77 x 10^(−10)	0.96 (0.93, 0.98)	4.82 x 10^(−4)
Magnesium	0.89 (0.87, 0.91)	1.51 x 10^(−19)	0.93 (0.90, 0.95)	2.18 x 10^(−9)	0.97 (0.95, 0.99)	0.016
Potassium	0.93 (0.91, 0.95)	8.97 x 10^(−9)	0.96 (0.94, 0.98)	0.001	0.97 (0.94, 0.99)	0.007
Starch	1.04 (1.02, 1.07)	6.33 x 10^(−4)	1.05 (1.02, 1.07)	3.12 x 10^(−4)	1.05 (1.02, 1.07)	2.32 x 10^(−4)
Vitamin E	0.92 (0.90, 0.94)	1.63 x 10^(−10)	0.95 (0.92, 0.97)	2.22 x 10^(−5)	0.97 (0.94, 0.99)	0.009

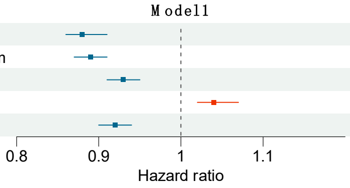

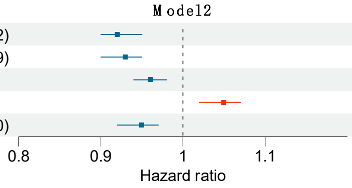

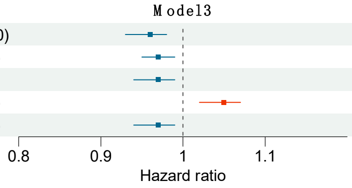

Figure S3. Forest plot for the association between significant dietary nutrient factors and T2DM risk (S3a) and the association between significant dietary nutrient factors and T2DM risk (S3b) excluding participants with nutrient intake among the top/bottom 1%. Abbreviation: HR, Hazard ratio; CI, confidence interval. The HR represents the increased T2DM risk for each quintile increase in dietary factor consumption. Model 1 is adjusted for gender and age at recruitment. Model 2 is additionally adjusted for ethnic background, smoking status, physical activity, education level, and Townsend index. Model 3 is further adjusted for BMI, HbA1c, blood cholesterol, medication for blood pressure and exogenous hormone, and medical condition of hypertension. For Model 2 and 3, non-alcoholic dietary food and nutrient variables were additionally adjusted for alcohol intake. Negative associations are depicted in blue, while positive associations are shown in red. Factors that are significant in both Model 1 and Model 2 will be highlighted in Model 2. Factors that are significant in all models will be highlighted in the Model 3 column.

Figure S4. Kaplan-Meier survival curves.

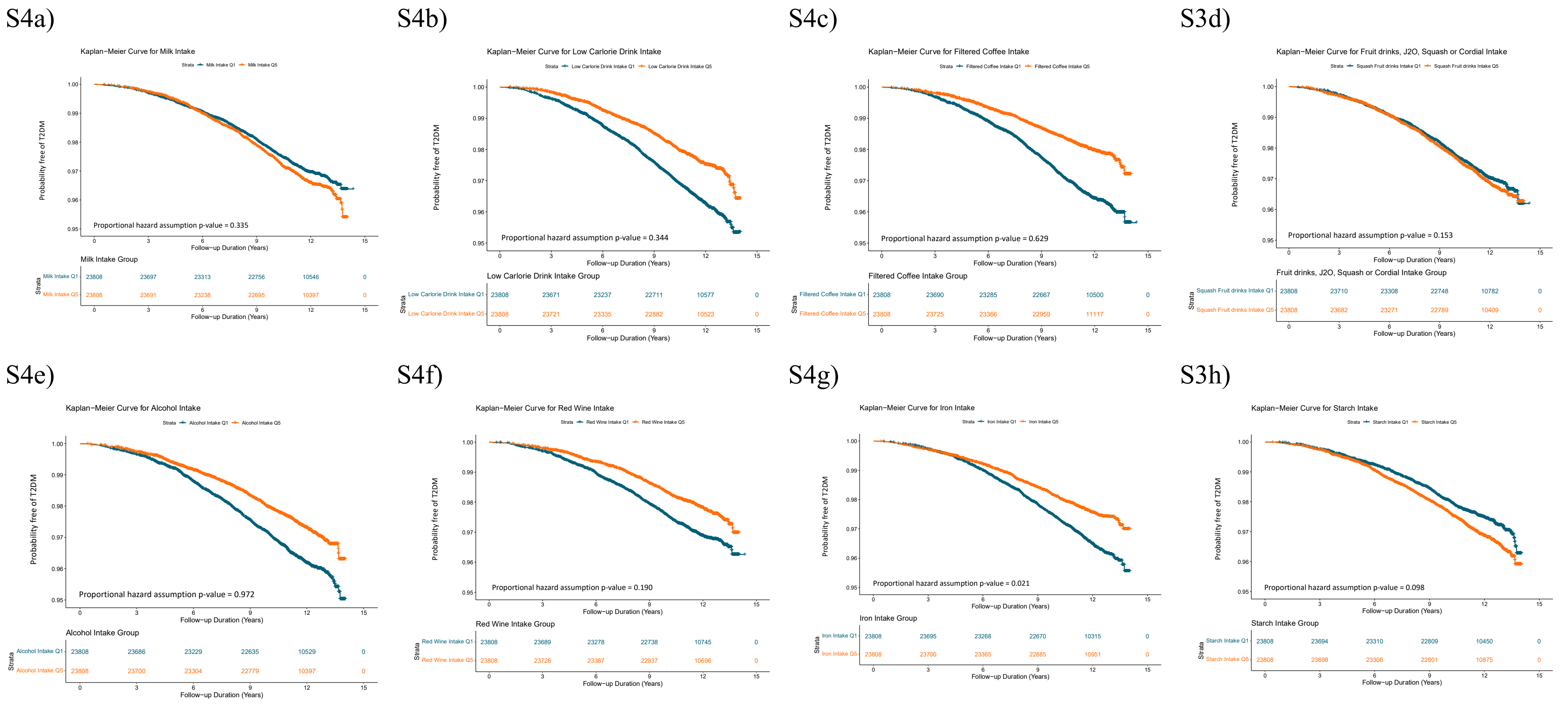

Figure S4. Kaplan-Meier survival curves for the onset of T2DM comparing the first and fifth quintiles of various dietary factors. Abbreviation: Q1, Quintile 1; Q5, Quintile 5. Only those dietary factors that are recorded in detail and not categorized by measurement units are shown. The x-axis represents the follow-up time in years, while the y-axis indicates the probability of remaining free from T2DM. The shaded areas represented the 95% confidence intervals. The table below the curves shows the number of participants at risk in different groups at various follow-up times.
